# Inclusion Complexes of *Litsea cubeba* (Lour.) Pers Essential Oil into β-Cyclodextrin: Preparation, Physicochemical Characterization, Cytotoxicity and Antifungal Activity

**DOI:** 10.3390/molecules29071626

**Published:** 2024-04-04

**Authors:** Giseli Cristina Pante, Juliana Cristina Castro, Renata Sano Lini, Jéssica Cristina Zoratto Romoli, Thiago Yoshioka Pires, Francielle Pelegrin Garcia, Celso Vataru Nakamura, Ana Claúdia Nogueira Mulati, Graciette Matioli, Miguel Machinski Junior

**Affiliations:** 1 Department of Health Basic Science, Laboratory of Toxicology, State University of Maringa, Avenue Colombo n° 5790, Maringa 87020-900, PR, Brazil; giselipante1994@gmail.com (G.C.P.); renatalini23@gmail.com (R.S.L.); jeczromoli@gmail.com (J.C.Z.R.); fpgarcia2@uem.br (F.P.G.); cvnakamura@uem.br (C.V.N.); mmjunior@uem.br (M.M.J.); 2 Department of Physics, State University of Maringa, Avenue Colombo n° 5790, Maringa 87020-900, PR, Brazilanacnogueira87@gmail.com (A.C.N.M.); 3 Department of Pharmacy, State University of Maringa, Avenue Colombo n° 5790, Maringa 87020-900, PR, Brazil; gmatioli@uem.br

**Keywords:** molecular inclusion, *Litsea cubeba* essential oil, β-cyclodextrin, antifungal activity, cytotoxic effect

## Abstract

The uses of natural compounds, such as essential oils (EOs), are limited due to their instability to light, oxygen and temperature, factors that affect their application. Therefore, improving stability becomes necessary. The objective of this study was to prepare inclusion complexes of *Litsea cubeba* essential oil (LCEO) with β-cyclodextrin (β-CD) using physical mixing (PM), kneading (KN) and co-precipitation (CP) methods and to evaluate the efficiency of the complexes and their physicochemical properties using ATR-FTIR, FT-Raman, DSC and TG. The study also assessed cytotoxicity against human colorectal and cervical cancer cells and antifungal activity against *Aspergillus flavus* and *Fusarium verticillioides*. The complexation efficiency results presented significant evidence of LCEO:β-CD inclusion complex formation, with KN (83%) and CP (73%) being the best methods used in this study. All tested LCEO:β-CD inclusion complexes exhibited toxicity to HT-29 cells. Although the cytotoxic effect was less pronounced in HeLa tumor cells, LCEO-KN was more active against Hela than non-tumor cells. LCEO-KN and LCEO-CP inclusion complexes were efficient against both toxigenic fungi, *A. flavus* and *F. verticillioides*. Therefore, the molecular inclusion of LCEO into β-CD was successful, as well as the preliminary biological results, evidencing that the β-CD inclusion process may be a viable alternative to facilitate and increase future applications of this EO as therapeutic medication, food additive and natural antifungal agent.

## 1. Introduction

Essential oils (EOs) are natural mixtures of volatile chemical compounds. They are synthesized using medicinal and aromatic plants as secondary metabolites. The EOs are known worldwide for their potent biological properties, which are attributed to chemical constituents, mainly terpenoids and phenolic compounds. Furthermore, the production of EOs is a process that occurs in almost all plant organs, particularly in the form of flowers, buds, leaves, seeds, stems and fruits [1].

*Litsea cubeba* (Lour.) Pers (Lauraceae family) is a significant medicinal plant that is widely distributed in China, Japan and Southeast Asian countries. It is commonly referred to as May Chang or Chinese pepper [2]. The *L. cubeba* essential oil (LCEO) extracted from fresh fruits has a citral content of approximately 60–90% [2,3]. In fact, several researchers have documented the bioactive properties of LCEO, which include antioxidant, antimicrobial, antifungal, anti-inflammatory and insecticidal properties [4,5,6,7].

The applications of EOs are limited due to their instability to light, oxygen and temperature [3]. Thus, an improvement in the stability of EOs is necessary to expand their applications in the food, cosmetic and medicine industries. In this context, alternative strategies, such as the inclusion of EOs into macromolecules, have been developed. Among them, cyclodextrins are cyclic oligosaccharides with a truncated-cone shape containing glucopyranose units. The most used cyclodextrin is β-cyclodextrin (β-CD), which contains seven glucose units, a hydrophobic cavity and a hydrophilic external surface [8].

The preparation of β-CD inclusion complexes involves numerous methods, such as solvent evaporation, freeze-drying, co-precipitation, physical mixture and kneading [9,10,11,12]. The literature reveals that the employed method has a significant effect on the formation of the inclusion complex [12].

Recently, many studies on the complexation of β-CD with EOs have been reported for different purposes. Galvão et al. [9] reported inclusion complexes of *Citrus sinensis* EO in β-CD on *Aedes aegypti* larvae. The *E. brejoensis* EO inclusion complex with β-CD exhibited cytotoxic activity against Hela and J774 cells [13]. Halahlah et al. [14] discussed the antibacterial activity of β-CD inclusion complexes containing *Rosmarinus officinalis* EO.

Furthermore, biological properties of inclusion complexes between LCEO and β-CD have been studied, such as antibacterial activity against *Staphylococcus aureus* [15] and antifungal activity on *Penicillium italicum*, *P. digitatum* and *Geotrichum citri-aurantii* [16]. However, to the best of our knowledge, there are still no studies on the effect of LCEO:β-CD inclusion complex on the cytotoxicity against human colorectal and cervical cancer cells, as well as on antifungal activity against *Aspergillus flavus* and *Fusarium verticillioides*.

Since there are no studies on the complexation of β-CD and LCEO, the aim of this research was to prepare inclusion complexes of LCEO in β-CD using physical mixture, kneading and co-precipitation methods. Moreover, the inclusion complexes were characterized in terms of physicochemical properties, cytotoxic effect and antifungal activity.

## 2. Results and Discussion

### 2.1. Complexation Efficiency (CE)

The complexation efficiency (CE%) was evaluated to quantify the amount of active compound of LCEO entrapped in the inclusion complex with β-CD [16]. In our study, the CE% showed different percentages (*p* < 0.01) for the LCEO:β-CD inclusion complexes using physical mixture (PM), kneading (KN) and co-precipitation (CP) methods, suggesting that the amount of the active substance was entrapped in the inclusion complex [16]. The LCEO-KN complex had a superior value compared to the other complexes, corresponding to 83%. The second-best method was LCEO-CP, with 73% efficiency, followed by LCEO-PM, with 48%. These results indicate good efficiency for the studied inclusion complexes, proving that the LCEO initially added to the process remained in the complexes obtained.

Halahlah et al. [14] reported CE% for the inclusion complexes of *R. officinalis*: β-CD using different methods in the range of 36 to 78%. According to the authors, these results can be attributed to the chemical structure and physical properties of β-CD and R. officinalis EO. These results agreed with those obtained in our study. In contrast, Wang et al. [16] showed a lower CE% result for the LCEO:β-CD inclusion complex using the saturated aqueous solution method, corresponding to 34%.

### 2.2. Physicochemical Properties of Inclusion Complexes

#### 2.2.1. Attenuated Total Reflection Fourier Transform Infrared Spectroscopy (ATR-FTIR)

**Figure 1:**
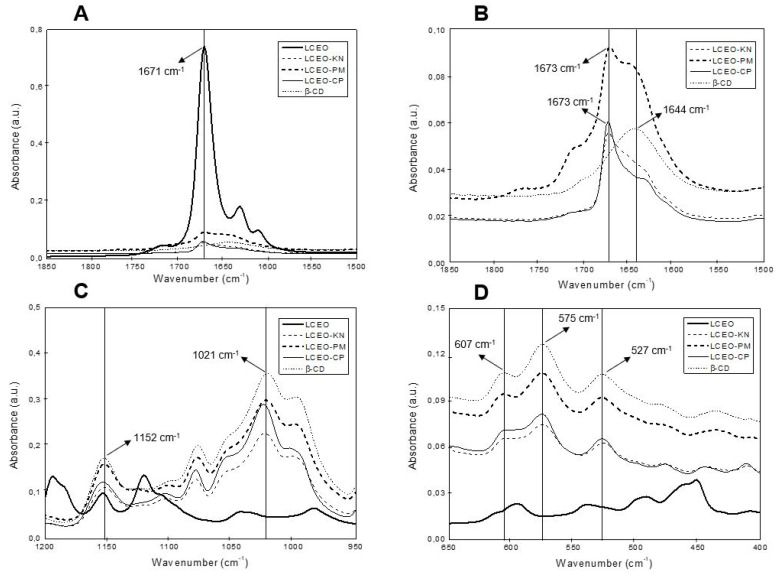
ATR-FTIR spectra of β-cyclodextrin (β-CD), *Litsea cubeba* essential oil (LCEO) and the inclusion complexes of LCEO in β-CD obtained from kneading (KN), co-precipitation (CP) and physical mixture (PM) methods. (**A**,**B**) Wavenumber from 1850 to 1500 cm^−1^; (**C**) Wavenumber from 1200 to 950 cm^−1^; (**D**) Wavenumber from 650 to 400 cm^−1^.

ATR-FTIR was used for the characterization of the inclusion complexes of LCEO:β-CD through different methods (kneading, co-precipitation and physical mixture) based on the comparison between the position and intensity of the spectral peaks obtained for LCEO, β-CD and LCEO:β-CD. ATR-FTIR and FT-Raman are complementary spectroscopic techniques used to characterize and prove the complexation between LCEO and β-CD. Figure 1 presents the most important regions of ATR-FTIR spectra of β-CD, LCEO and LCEO:β-CD inclusion complexes produced through different methods.

In the ATR-FTIR spectra of β-CD (Figure 1B–D), several characteristic absorption peaks were observed. The peak at 1644 cm^−1^ (Figure 1B) represents the deformation mode of crystallization water present in the β-CD cavity [14]. In addition, the absorption peak at 1152 cm^−1^ (Figure 1C) was related to C-O stretching vibrations and O-H bending, while the band detected at 1021 cm^−1^ resulted from the stretching vibration of C-O and C-C groups [17]. Finally, the peaks at 607 cm^−1^, 575 cm^−1^ and 527 cm^−1^ (Figure 1D) represent the out-of-plane bending vibrations of OH [18].

For LCEO (Figure 1A), an important band was registered at 1671 cm^−1^, which was related to the C=O stretching vibration of the citral compound, the major constituent of LCEO [16]. Nevertheless, for all inclusion complexes, this vibration shifted from 1671 cm^−1^ to 1673 cm^−1^, and the peak intensity decreased. According to Răileanu et al. [17], the displacement of bands or changes in the intensity of the ATR-FTIR signals are indicative of the interaction between β-CD and EO. This result suggests the synthesis of LCEO:β-CD inclusion complexes.

The interaction between essential oil and β-CD is reported in the literature, with the following chemical interactions: hydrophobicity, hydrogen bonding, the release of high-energy water molecules from β-CD cavities, and Van der Waals forces [10]. These chemical interactions indicate the interaction between the guest components of LCEO and the host molecule of β-CD.

#### 2.2.2. Fourier Transform Raman Spectroscopy (FT-Raman)

**Figure 2:**
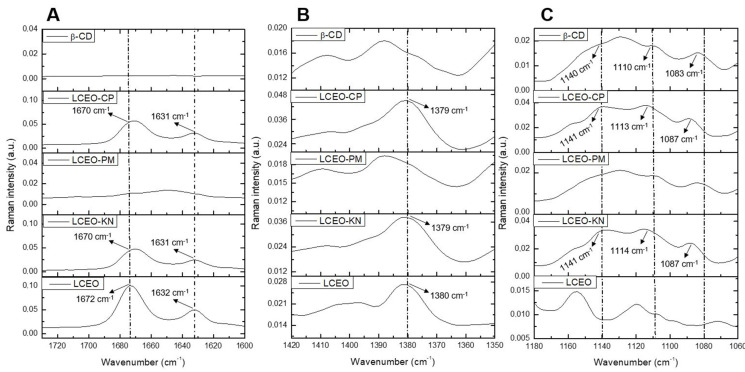
Raman spectra of β-cyclodextrin (β-CD), *Litsea cubeba* essential oil (LCEO) and the inclusion complexes of LCEO in β-CD obtained from kneading (KN), co-precipitation (CP) and physical mixture (PM) methods. (**A**) Wavenumber from 1720 to 1600 cm^−1^; (**B**) Wavenumber from 1420 to 1350 cm^−1^; (**C**) Wavenumber from 1180 to 1060 cm^−1^.

The important regions of the Raman spectra for β-CD, LCEO and LCEO:β-CD inclusion complexes produced through different methods are shown in Figure 2. The main chemical compounds previously obtained via GC-MS for LCEO were geranial and neral, with 37.67% and 32.75%, respectively [6]. Both compounds are geometric isomers and aliphatic unsaturated aldehydes, so the expected signals for FT-Raman are related to the C=O and C=C bonds [19].

In the Raman spectra of LCEO (Figure 2A), a strong absorption band appeared at 1672 cm^−1^, which was assigned to the stretching vibration of the C=O group in the citral compound [19]. In LCEO-CP and LCEO-KN inclusion complexes, this vibration shifted from 1672 cm^−1^ to 1670 cm^−1^. Likewise, the C=C stretching mode was detected at 1632 cm^−1^ for LCEO and shifted to 1631 cm^−1^ in the LCEO-CP and LCEO-KN inclusion complexes. Moreover, the peak intensity decreased, indicating that LCEO was successfully included in the cavity of β-CD.

The band obtained at 1380 cm^−1^ for the LCEO (Figure 2B) was also observed in LCEO-CP and LCEO-KN inclusion complexes. Vibration was favored after the interaction of the LCEO with β-CD. Meanwhile, LCEO-PM was similar to β-CD. Hanif et al. [20] attributed the 1378 cm^−1^ wavenumber to the compound limonene, which is present in the chemical composition of LCEO with 10.55%.

The FT-Raman spectra of β-CD (Figure 2C) showed prominent absorption bands at 1083 cm^−1^ for C-O stretching vibration, 1110 cm^−1^ for C-C stretching vibration and 1140 cm^−1^ for C-H scissoring vibration [18]. When comparing the β-CD spectra with the LCEO-CP and LCEO-KN inclusion complexes spectra, we could observe the displacement of β-CD at these wavenumbers, which indicates the formation of the inclusion complexes. On the other hand, the Raman spectra of LCEO-PM (Figure 2A–C) were similar to β-CD, evidencing little or no interaction between LCEO and β-CD in this inclusion complex.

The ATR-FTIR and FT-Raman spectroscopic techniques were effective in evaluating molecular vibrations in complex samples of both β-CD and LCEO compounds [20]. Based on the results of ATR-FTIR (Figure 1) and FT-Raman (Figure 2), we highlight the interaction of the citral functional group in the 1600 cm^−1^ region for both techniques. This same result has been reported by other authors, reinforcing the interaction of this functional group in the hydrophobic cavity of β-CD [16,19]. Thus, it could be inferred that the components of LCEO entered the hydrophobic cavity of β-CD through co-precipitation (LCEO-CP) and kneading (LCEO-KN) methods.

#### 2.2.3. Thermal Analysis

**Figure 3:**
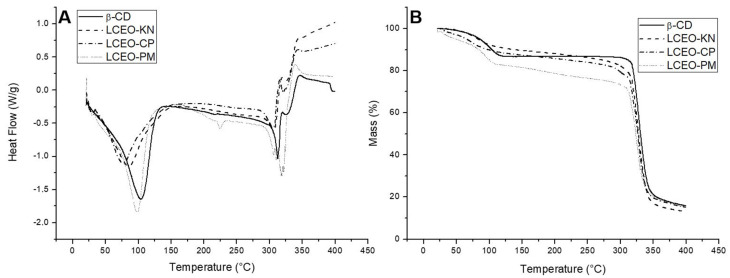
DSC (**A**) and TG (**B**) thermograms in dynamic N_2_ atmosphere of β-cyclodextrin (β-CD) and the inclusion complexes of *Litsea cubeba* essential oil (LCEO) in β-CD obtained from kneading (KN), co-precipitation (CP) and physical mixture (PM) methods.

Thermal analyses were performed to confirm the formation of the inclusion complexes. Figure 3 illustrates the DSC and TG thermograms of β-CD and LCEO:β-CD inclusion complexes produced through different methods.

The DSC curve of β-CD (Figure 3A) showed endothermic peaks at temperatures of 104 °C and 314 °C. According to the literature, the first peak indicates the release of water molecules, while the second peak corresponds to the melting point [9,21]. In addition, exothermic peaks were observed around 321 °C and 346 °C, attributed to the decomposition of β-CD. Likewise, Galvão et al. [9] and Miyoshi et al. [10] reported that peaks above 300 °C correspond to decomposition and removal of carbonaceous material.

The DSC thermogram of LCEO-PM (Figure 3A) was similar to β-CD, indicating little or no interaction between LCEO and β-CD in this inclusion complex. However, when comparing the DSC curves of LCEO-KN and LCEO-CP inclusion complexes to β-CD, we could observe variations in both intensities and temperatures. For LCEO-KN (Figure 3A), the temperatures found were 83 °C, 307 °C and 341 °C, and for LCEO-CP (Figure 3A), they were 80 °C, 305 °C and 343 °C, thus providing an indication of the interaction between components of LCEO and β-CD in these inclusion complexes.

The TG thermogram of β-CD (Figure 3B) showed two stages of thermal degradation. The initial mass loss phase occurred at the range of 85–130 °C, which was possibly caused by water evaporation, while the second weight loss was observed in the range of 310–370 °C, probably attributed to the molecular decomposition of β-CD. Our observations were consistent with those found in the literature [15,16].

For the LCEO-PM (Figure 3B) inclusion complex, the TG curve was similar to β-CD, with initial and secondary mass losses distributed in the range of 90–125 °C and 300–370 °C, respectively. Both LCEO-KN and LCEO-CP (Figure 3B) inclusion complexes presented only one thermal degradation stage, which was relatively slow and stable until the molecular decomposition of β-CD, in the range of 290–370 °C. In addition, the water loss (85–130 °C) was lower than β-CD. Wang et al. [16] attributed this observation to the hydrophobic interaction between EO and β-CD. Therefore, LCEO-KN and LCEO-CP were more thermally stable than LCEO-PM.

### 2.3. Cytotoxicity of Inclusion Complexes

The LCEO:β-CD inclusion complexes obtained through different methods were tested against tumor (HT-29 and HeLa) and non-tumor (Vero) cell lines to evaluate their cytotoxic effect. The results of the MTT assay are presented in Table 1. In summary, most of the inclusion complexes of LCEO into β-CD exhibited antitumor activity against the investigated cells. Moreover, cell viability was not affected in cultures treated with β-CD (IC_50_ > 1000 µg mL^−^^1^).

In this study, the two cell cultures tested are among the 10 most prevalent types of cancer in the world. Interestingly, the inclusion complexes showed to be more selective against HT-29 tumor cell (IC_50_ = 71.5–81.7 µg mL^−1^) since the IC_50_ obtained for this cell was lower than that obtained for Vero cell (IC_50_ = 88.0–99.0 µg mL^−1^). So, all tested LCEO:β-CD inclusion complexes exhibited toxicity to HT-29 cells. Moreover, it was observed that the cytotoxic effect of inclusion complexes was less pronounced in HeLa tumor cells (IC_50_ = 88.0–106.3 µg mL^−1^); however, LCEO-KN was also more active against Hela than the non-tumor cell.

Previous studies have demonstrated that EOs and their isolated compounds are associated with antitumor activity in different carcinogenic cells, such as *Laurus nobilis* EO on K562 human chronic myelogenous leukemia cells [22] and *E. brejoensis* EO against J774 and HeLa tumor cells [13], as well as isomers of citral [23], the same compounds found in LCEO. Furthermore, Santana et al. [13] described the cytotoxicity of the *E. brejoensis* EO into β-CD against J774 and HeLa cell lines.

### 2.4. Antifungal Activity of Inclusion Complexes

The agar dilution method was used to evaluate the inhibitory capacity of LCEO and its inclusion complexes against *A. flavus* and *F. verticillioides*. The results are shown in Table 2. In general, the inclusion complexes significantly inhibited radial growth compared to LCEO and FC (*p* < 0.05) and exhibited good antifungal activity. In addition, no radial inhibition was observed for the isolated β-CD.

Lower effectiveness of LCEO against *A. flavus* and *F. verticillioides* was observed, followed by the LCEO-PM inclusion complex. In contrast, the LCEO-KN and LCEO-CP inclusion complexes were more efficient for both toxigenic fungi evaluated. These findings confirm that the antifungal activity of LCEO was improved after molecular inclusion into β-CD. Thus, the molecular inclusion of LCEO into β-CD has potential application as a natural fungicide in the control of *A. flavus* and *F. verticillioides*.

Our observations were consistent with those found in previous studies [14,15]. Wang et al. [13] evidenced a good antifungal activity of the inclusion complex of LCEO with β–CD against *P. italicum*, *P. digitatum* and *G. citri-aurantii* isolated from postharvest citrus. Although there are no studies indicating the antifungal efficacy of LCEO complexed with β–CD against the toxigenic fungi *A. flavus* and *F. verticillioides*, in these studies, the molecular inclusion of EOs improved the antimicrobial and antifungal activities, possibly due to the greater water solubility of the inclusion complexes compared to pure EO. More studies are needed to better understand the antifungal mechanisms of LCEO and its inclusion complex against toxigenic fungi.

## 3. Materials and Methods

### 3.1. Chemicals

The LCEO (By Samia^®^, Cotia, Brazil) was acquired in a market located in Maringa City, Parana State, Brazil, in a single lot (number: 217). Ethanol was purchased from Synth^®^ (Diadema, Brazil), and potato dextrose agar medium (PDA) was obtained from Himedia^®^ (Mumbai, India). β-CD, Tween-80, 3-(4,5-dimethyl-2-thiazolyl)-2,5-diphenyl-2*H*-tetrazolium bromide (MTT), L-glutamine, phosphate-buffered saline (PBS) and dimethyl sulphoxide (DMSO) were obtained from Sigma-Aldrich^®^ (St. Louis, MO, USA). Dulbecco’s modified Eagle’s medium (DMEM) and fetal bovine serum (FBS) were obtained from Gibco Invitrogen^®^ (New York, NY, USA). All reagents were of analytical grade.

### 3.2. Preparation of Inclusion Complexes

The inclusion complexes of LCEO and β-CD were prepared in a molar ratio of 1:1, based on the molecular weight of citral (152.24 g mol^−1^), using the physical mixture, kneading and co-precipitation methods.

The citral chemical compound was the major constituent of LCEO, previously determined through gas chromatography coupled with mass spectrometry (GC-MS), accounting for 70.42% (32.75% neral and 37.67% geranial), followed by limonene with 10.55% of the total EO [6]. The material was stored in hermetically sealed amber glass flasks and stored at −20 °C until analysis. All analyses were conducted in triplicate.

#### 3.2.1. Physical Mixture

A physical mixture (PM) was obtained by the addition of LCEO to a glass mortar containing β-CD under manual agitation and stored in amber glass containers until the moment of analysis [9].

#### 3.2.2. Kneading

For the kneading (KN), β-CD and the LCEO were homogenized in a glass mortar. Then, a mixture of distilled water and ethanol (1:1) was progressively added until a paste formed. The resulting material was dried in a desiccator, which was removed through manual trituration and stocked in amber glass containers for further measurements [9].

#### 3.2.3. Co-Precipitation

In the co-precipitation (CP) method, β-CD was solubilized in 40 mL of distilled water in a water bath at 60 °C. The solution was cooled to 25 °C, and the LCEO dissolved in ethanol was slowly added. Then, the sample was stirred at 140 rpm and 25 °C for 60 min and subjected to vacuum filtration. The resulting material was dried in a desiccator and stored in amber glass containers for further analysis [9].

### 3.3. Complexation Efficiency (CE)

To determine the LCEO content in the inclusion complexes, 1 mg of the EO and complexes were diluted in 2 mL of ethanol and then filtered through 0.45 μm PTFE filters. The absorbances of the samples were determined through a UV spectrophotometer (Shimadzu UV 1601 PC, Columbia, SC, USA) at a wavelength of 216 nm. For each measurement, ethanol was used as a reference blank [11]. The complexation efficiency (CE%) was calculated as follows:

(1) $$\mathrm{CE}\%=\left(\frac{L_{c}}{L_{t}}\right)\times 100,$$

where L_c_ is the mass of the complexed EO, and L_t_ is the total mass of EO added initially.

### 3.4. Physicochemical Properties of Inclusion Complexes

#### 3.4.1. Attenuated Total Reflection Fourier Transform Infrared Spectroscopy (ATR-FTIR)

The ATR-FTIR spectra of LCEO, β-CD and inclusion complexes (PM, KN and CP) were obtained on a Vertex 70v Spectrometer (Brucker, Karlsruhe, Germany) with a device for attenuated reflectance (Platinum ATR, Bruker, Germany). Spectra were recorded without any sample preparation. The spectral range was 400–4000 cm^−1^ with 128 scans at 4 cm^−1^ resolution [11,17].

#### 3.4.2. Fourier Transform Raman Spectroscopy (FT-Raman)

The FT-Raman spectra of LCEO, β-CD and inclusion complexes (PM, KN and CP) were measured using an infrared Fourier transform Spectrometer (model Vertex 70v with Ram II module, Bruker, Germany) equipped with a liquid nitrogen-cooled Germanium detector. Spectra were recorded at wavelengths between 400 and 4000 cm^−1^ without any sample preparation. A Nd:YAG laser was used for excitation at 1064 nm with 70 mV. All of the spectra were an average of 128 scans with a 4 cm^−1^ resolution [11].

#### 3.4.3. Thermal Analysis

The differential scanning calorimetry (DSC) and thermogravimetry (TG) analyses were performed on a thermal analyzer (model STA 409 PG LUXX, Netzsch, Selb, Germany) involving samples of β-CD and inclusion complexes (PM, KN and CP) under a N_2_ volumetric flow of 30 mL min^−1^ at atmospheric pressure. The temperature varied between 0 °C and 400 °C at a heating rate of 10 °C min^−1^ [24].

### 3.5. Cytotoxicity of Inclusion Complexes

#### 3.5.1. Cell Cultures

We used 3 cell ATTC lines: HT-29 (ATCC HTB-38) as human colorectal adenocarcinoma, HeLa (Cervix adenocarcinoma—ATCC CCL-2) as cervical cancer and Vero (ATCC CCL-81—African green monkey border epithelial cells) as normal cell line (control), was obtained from the American Type Culture Collection (ATCC, Manassas, VA, USA 30-4500 K). The cell cultures were performed (2.5 × 10^5^ cells mL^−1^) using DMEM supplemented with 10% FBS and 2 mM L-glutamine. All cell lines were dispensed into a sterile 96-well plate and incubated for 24 h at 37 °C in a humidified atmosphere with 5% CO_2_.

#### 3.5.2. MTT Assay

The cytotoxicity was performed using the MTT assay according to Mosmann’s protocol [25]. After the cell culture period, the supernatant was withdrawn, and increasing concentrations of β-CD and inclusion complexes (PM, KN and CP) were added (0 to 1000 µg mL^−1^). After 48 h of incubation under the same culture conditions, the cells were washed with 100 μL of 0.01 M PBS, and 50 μL of MTT at a concentration of 2 mg mL^−1^ was added, followed by incubation for 4 h at 37 °C. Formazan crystals were solubilized in DMSO, and the absorbance was evaluated in a spectrophotometer (BioTek Power Wave XS, Winooski, VT, USA) at 570 nm. The experiment was performed in triplicate, and the cytotoxic activity was expressed as the concentration of the sample that inhibited 50% of cell growth compared to the control (IC_50_).

### 3.6. Antifungal Activity of Inclusion Complexes

#### 3.6.1. Microorganisms

The strains of *A. flavus* (AF42) and *F. verticillioides* (103F) were obtained from the collection of Toxicology Laboratory, State University of Maringa, Brazil. *A. flavus* was cultivated in a PDA medium at 25 °C for 7 days, without natural or artificial light, in a biological oxygen demand (BOD) incubator (Ethik Technology, Vargem Grande Paulista, Brazil). *F. verticillioides* was cultured in a PDA medium at 25 °C for 15 days and exposed to black light (26 W, 3 U, 127 V) in a BOD incubator.

#### 3.6.2. Agar Dilution Method

The LCEO and inclusion complexes (PM, KN and CP) were dissolved in 1% Tween-80 at a concentration of 1000 µg mL^−1^ and added to the PDA medium at a temperature of 40–45 °C, then poured into Petri dishes (90 mm). The fungi were inoculated as soon as the medium had solidified. Discs of mycelial (8 mm) from *A. flavus* and *F. verticillioides*, taken from the edge of seven- and fourteen-day-old fungal cultures, respectively, were placed at the center of each Petri dish. The fungal controls were prepared similarly by inoculating only the mycelial discs. The Petri dishes were placed in a BOD incubator under controlled temperature conditions of 25 °C, without light to *A. flavus* and with black light for *F. verticillioides*. The efficacy of treatments was evaluated after 7 days in triplicate [26]. The percentage of radial inhibition (RI%) was calculated as follows:

(2) $$\mathrm{RI}\%=\left(\frac{L_{c}-L_{t}}{L_{c}}\right)\times 100$$

where L_c_ (cm) is the mean of radial growth for the fungal controls, and L_t_ (cm) is the mean of radial growth for each group treated with the LCEO and inclusion complexes.

### 3.7. Statistical Analysis

Data were evaluated using one-way analysis of variance (ANOVA), followed by the Tukey test using BioEstat 5.3 software (Mamirauá Institute, Tefé, AM, Brazil). The graphs were generated using the software SigmaPlot 11.0 (Systat, San Jose, CA, USA) and Origin 8.0 (Originlab, Northampton, MA, USA).

## 4. Conclusions

The complexation efficiency, ATR-FTIR, FT-Raman, DSC and TG results presented significant evidence of LCEO:β-CD inclusion complex formation. These results indicated that kneading and co-precipitation were the best methods used in this study. We have demonstrated that the method of preparation can have a significant impact on the formation of the inclusion complex due to the effectiveness of encapsulation observed in the physical mixture. The molecular inclusion of components of LCEO into β-CD was successful, as well as the preliminary biological results, evidencing that the β-CD inclusion process may be a viable alternative to facilitate and increase future applications of this EO as a therapeutic medication and natural antifungal agent.

## Figures and Tables

**Table 1:** Antitumor activity and cytotoxicity of β-cyclodextrin (β-CD) and the inclusion complexes of *Litsea cubeba* essential oil (LCEO) in β-CD obtained from kneading (KN), co-precipitation (CP) and physical mixture (PM) methods.

Samples	IC_50_ (ug mL^−1^)		
	HT-29	HeLa	Vero
β-CD	>1000	>1000	>1000
LCEO-KN	81.7 ± 7.5	88.0 ± 20.8	99.0 ± 11.5
LCEO-CP	74.8 ± 20.9	95.0 ± 21.2	88.0 ± 5.4
LCEO-PM	71.5 ± 5.6	106.3 ± 11.0	90.0 ± 2.6

IC_50_: Inhibitory Concentration for 50% of the cell. The values are the mean ± standard deviation for triplicates.

**Table 2:** Antifungal activity of β-cyclodextrin (β-CD), *Litsea cubeba* essential oil (LCEO) and the inclusion complexes of LCEO in β-CD obtained from kneading (KN), co-precipitation (CP) and physical mixture (PM) methods.

Samples	Radial Inhibition (%)	
	*Aspergillus flavus*	*Fusarium verticillioides*
FC	0 ^c^ ± 0	0 ^d^ ± 0
β-CD	0 ^c^ ± 0	0 ^d^ ± 0
LCEO	10.22 ^b^ ± 1.88	8.55 ^c^ ± 2.43
LCEO-KN	25.68 ^a^ ± 2.44	27.41 ^a^ ± 2.96
LCEO-CP	23.92 ^a^ ± 0.80	23.04 ^a, b^ ± 1.61
LCEO-PM	11.07 ^b^ ± 2.25	16.20 ^b^ ± 2.16

FC: fungal control (inoculum 10^5^ conidia mL^−^^1^). The values are the mean ± standard deviation for triplicates. Different letters between lines refer to significant differences (*p* < 0.05) using the Tukey test.

## Data Availability

Data are contained within the article.
